# Two-year longitudinal survey reveals high genetic diversity of *Schistosoma mansoni* with adult worms surviving praziquantel treatment at the start of mass drug administration in Uganda

**DOI:** 10.1186/s13071-019-3860-6

**Published:** 2019-12-27

**Authors:** Christina L. Faust, Marco Crotti, Arinaitwe Moses, David Oguttu, Aidah Wamboko, Moses Adriko, Elizabeth K. Adekanle, Narcis Kabatereine, Edridah M. Tukahebwa, Alice J. Norton, Charlotte M. Gower, Joanne P. Webster, Poppy H. L. Lamberton

**Affiliations:** 1 Institute of Biodiversity, Animal Health and Comparative Medicine, University of Glasgow, Glasgow, UK; 2 Wellcome Centre for Integrative Parasitology, University of Glasgow, Glasgow, UK; 3 Vector Control Division, Ministry of Health, Kampala, Uganda; 4 Department of Infectious Disease Epidemiology, Imperial College London, London, UK; 5 Department of Pathobiology and Population Sciences, Royal Veterinary College, Hawkshead, UK

**Keywords:** Schistosomiasis, Mass drug administration, Praziquantel, Trematodes, Population genetics, Interventions, Natural variation

## Abstract

**Background:**

A key component of schistosomiasis control is mass drug administration with praziquantel. While control interventions have been successful in several endemic regions, mass drug administration has been less effective in others. Here we focus on the impact of repeated praziquantel treatment on the population structure and genetic diversity of *Schistosoma mansoni*.

**Methods:**

We examined *S. mansoni* epidemiology, population genetics, and variation in praziquantel susceptibility in parasites isolated from children across three primary schools in a high endemicity region at the onset of the Ugandan National Control Programme. Children were sampled at 11 timepoints over two years, including one week and four weeks post-praziquantel treatment to evaluate short-term impacts on clearance and evidence of natural variation in susceptibility to praziquantel.

**Results:**

Prevalence of *S. mansoni* was 85% at baseline. A total of 3576 miracidia larval parasites, isolated from 203 individual children, were genotyped at seven loci. Overall, genetic diversity was high and there was low genetic differentiation, indicating high rates of parasite gene flow. Schistosome siblings were found both pre-treatment and four weeks post-treatment, demonstrating adult worms surviving treatment and natural praziquantel susceptibility variation in these populations at the beginning of mass drug administration. However, we did not find evidence for selection on these parasites. While genetic diversity decreased in the short-term (four weeks post-treatment), diversity did not decrease over the entire period despite four rounds of mass treatment. Furthermore, within-host genetic diversity was affected by host age, host sex, infection intensity and recent praziquantel treatment.

**Conclusions:**

Our findings suggest that praziquantel treatments have short-term impacts on these parasite populations but impacts were transient and no long-term reduction in genetic diversity was observed. High gene flow reduces the likelihood of local adaptation, so even though parasites surviving treatment were observed, these were likely to be diluted at the beginning of the Ugandan National Control Programme. Together, these results suggest that MDA in isolation may be insufficient to reduce schistosome populations in regions with high genetic diversity and gene flow.
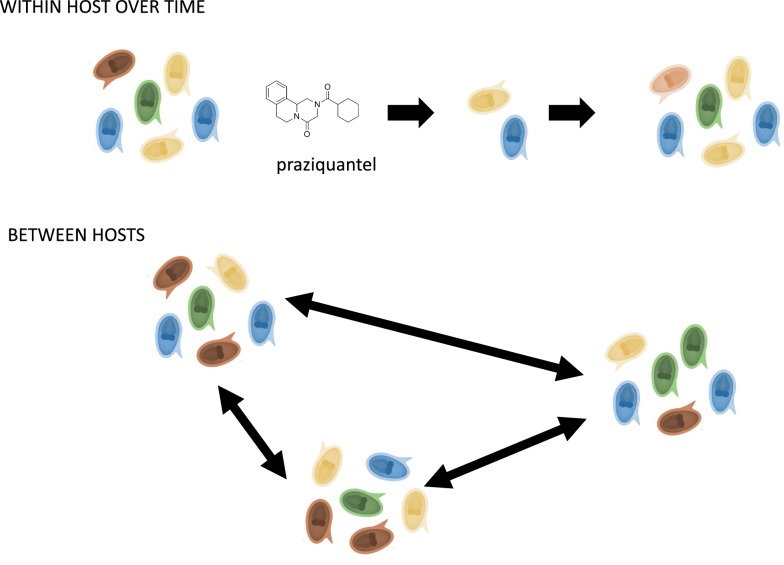

## Background

Schistosomiasis is a neglected tropical disease that infects over 240 million people across 78 countries, predominantly within the developing world [1]. Adult *Schistosoma mansoni* sexually reproduces (predominantly) in humans and eggs are excreted in faeces. In high endemicity areas, worm burdens can be very heavy, producing as many as 9600 eggs per gram (epg) of stool [2]. In areas with inadequate containment of stool due to poor sanitation, eggs contact freshwater and hatch into free-swimming miracidia. Miracidia then infect suitable snail intermediate hosts and undergo asexual reproduction, releasing thousands of free-swimming clonal cercariae daily [3]. Cercariae burrow through the skin to infect humans when they contact infectious water, through activities such as bathing, gathering water or fishing. Despite the integral role of insufficient water, sanitation and hygiene (WASH) in maintaining transmission, preventative chemotherapy through mass drug administration (MDA) with praziquantel is currently the main strategy for controlling morbidity, and ultimately transmission, of schistosomiasis in endemic areas [4]. While MDA has been successful in reducing morbidity and prevalence or intensity of schistosomiasis across many parts of sub-Saharan Africa [5–7], persistent transmission hotspots of *Schistosoma* species remain [8, 9].

Studies investigating the genetic structure of *Schistosoma* populations and their response to MDAs have the ability to quantify the impact and potential limitations of MDAs [10, 11]. These findings could help identify parasite-specific characteristics contributing to persistent transmission. The impact of treatment on parasite populations depends on many factors including, but not exclusive to, population coverage, frequency of drug pressure, baseline levels of parasite genetic diversity [12, 13], and rates of parasite gene flow [14]. Drug selection has been linked to lower effective population sizes [15] and can reduce genetic diversity of parasites in the laboratory [16]. In the field, treatment generally reduces prevalence and intensity of parasites in targeted populations and individuals [17, 18], but can also measurably reduce transmission rates across the population, influencing infections in individuals beyond the treated group [19–21]. However, treatments can also select for reduced drug efficacy and/or increase in resistance in populations [22–25]. Therefore, it is important to understand how parasites are structured across the landscape, and within individuals, in order to monitor treatment impacts and manage for the potential emergence and spread of drug resistance.

Several studies in Africa support a lack of genetic population structure in *Schistosoma* species at relatively small scales, from within villages to between sites up to 60 km apart [26–30]. High rates of gene flow suggest there are minimal barriers for transmission, at least at these geographical scales. The only large-scale study to date, to the authors’ knowledge, which encompassed five African countries, also found little support for structure between geographically close sites but distinct parasite clusters at the country level [31]. However, in some parts of Brazil, gene flow has been observed to be limited, even between sites 6 km apart [32]. Human movement patterns and water flow have also been shown to facilitate parasite population structure between different boroughs within a single town [33]. Higher overall levels of genetic diversity and longer history of transmission of *S. mansoni* in East Africa relative to South America may contribute to these differences in population structure, but studies explicitly evaluating these hypotheses are lacking.

At least under laboratory conditions, praziquantel reduces the diversity of *S. mansoni* and drug resistance can be selected for in as few as six generations [34, 35]. Reduced drug efficacy has also been recorded in several endemic areas, including Uganda [24, 36], though the geographic spread of resistance has not yet been documented. There are no genetic markers for resistance or reduced susceptibility to praziquantel in any schistosomes and the mechanism of action for the drug is unknown, complicating understanding treatment failures. The effect of praziquantel treatment on *S. mansoni* genetic diversity in the field also offers conflicting results. Reduction of genetic diversity has been observed six months after a single praziquantel treatment in two schools in Tanzania [21, 37]. In contrast, studies in Kenya showed school-based praziquantel MDA did not reduce genetic diversity over a five-year period [2] and another study in Senegal showed no reduction in genetic diversity over two years [30]. Similarly, a study in Brazil demonstrated little differentiation between parasites isolated pre-treatment and four to six weeks post-treatment [38].

Here we focus on the structure and genetic diversity of *S. mansoni* at the beginning of the MDA in Uganda, the first schistosomiasis MDA programme in sub-Saharan Africa [39]. Few field studies to date have examined *Schistosoma* genetic diversity over short (less than one month) and medium-term (six months or more) follow-ups after praziquantel treatment. In this study, we use a unique longitudinal dataset to examine how repeated praziquantel treatments can affect schistosome populations. We examine evidence for adult worms surviving treatment, suggesting natural variation in tolerance or resistance to praziquantel treatment. We hypothesize that mean genetic diversity would decline immediately following praziquantel treatment but expect diversity to recover at longer time-scales because of high gene flow and high genetic diversity at the population level. We expect clearance of parasites to be high, as host populations were praziquantel-naïve and parasites had not undergone repeated rounds of praziquantel selection.

## Methods

### Parasite sampling

**Fig. 1:**
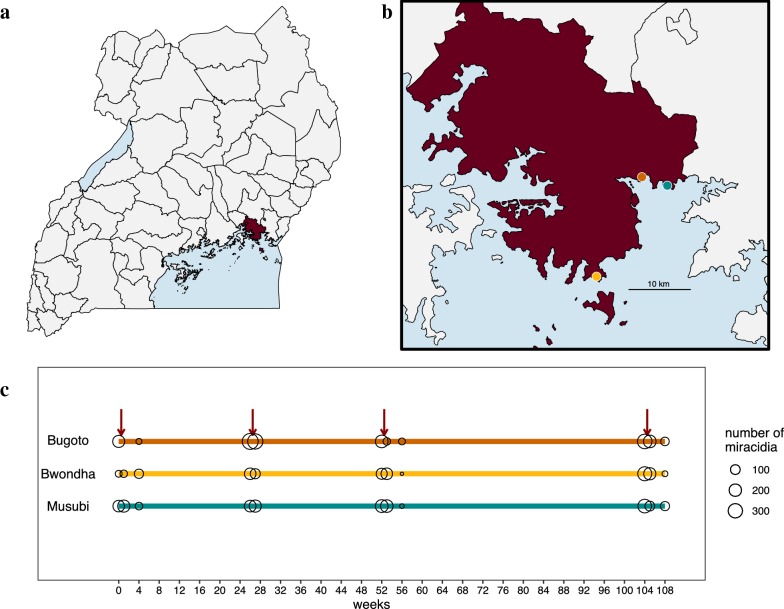
Sampling locations and timeline of genetic samples. **a** Map of Uganda with **b** Mayuge district (dark red) and the three primary schools [Bugoto Lake View (Bugoto-orange), Bwondha (Bwondha-yellow) and Musubi Church of God (Musubi-teal)] indicated and the number of miracidia sampled at each timepoint (**c**). The red arrows indicate praziquantel given to the entire cohort after medium-term schistosomiasis surveys

Children aged 6–12 years were initially recruited for this study in 2004 with an equal sex ratio from three primary schools on the shores of Lake Victoria in eastern Uganda (Fig. 1a, b). The primary schools are located in three separate villages within Mayuge District and between 4.35 km and 18.75 km apart (road and shoreline distances; as a proxy for actual travel distances are greater; Additional file 1: Table S1). Sample recruitment has been described previously [40, 41] and full details of new recruits and follow-ups are given in Additional file 1: Text S1 and Table S2. After the initial recruitment, an additional 30 praziquantel-naïve 6-year olds were recruited each year and included in follow-up surveys. Across a two-year period, there were a total of 11 sampling timepoints (Fig. 1c). Sample timepoints were designed to capture the effect of praziquantel treatment on parasite epidemiology and genetics in the short-term (one week and four weeks post-treatment) and medium-term (six months or more post-treatment). We acknowledge that medium-term is not reflective of an absolute definition but is used within this manuscript for convenience to describe discrete sampling windows.

At each timepoint, stool samples were collected for three consecutive days to measure infection intensity by duplicate Kato-Katz thick smears [42]. The number of *S. mansoni* eggs observed in a slide was multiplied by 24 to obtain eggs per gram (epg) (a standard 41.2 mg template was used to prepare). After Kato-Katz slides were prepared, the remainder of each stool sample was filtered through a Pitchford funnel to collect and hatch miracidia from eggs [43]. Filtered samples were exposed to sunlight and individual miracidia were picked up in 2.5–5.0 μl bottled spring water under a stereomicroscope. In 2004, single miracidia were placed in individual PCR tubes and kept cool until flash freezing each evening in a − 80 °C freezer and then shipped on dry ice to Imperial College London. From 2005 onwards, individual miracidia were placed on Whatman Indicating FTA^TM^ cards for cell lysis and DNA storage [44]. Cards were kept at room temperature in sealed plastic bags with desiccants in the field and during transport. As many miracidia as possible were collected for each child onto a single FTA card per timepoint resulting in a final range of 0–132 miracidia collected per child at any given timepoint. We use previous nomenclature and define all parasites isolated from a single child as an infrapopulation [37]. The cumulative number of miracidia at each timepoint is given in Fig. 1c.

**Fig. 2:**
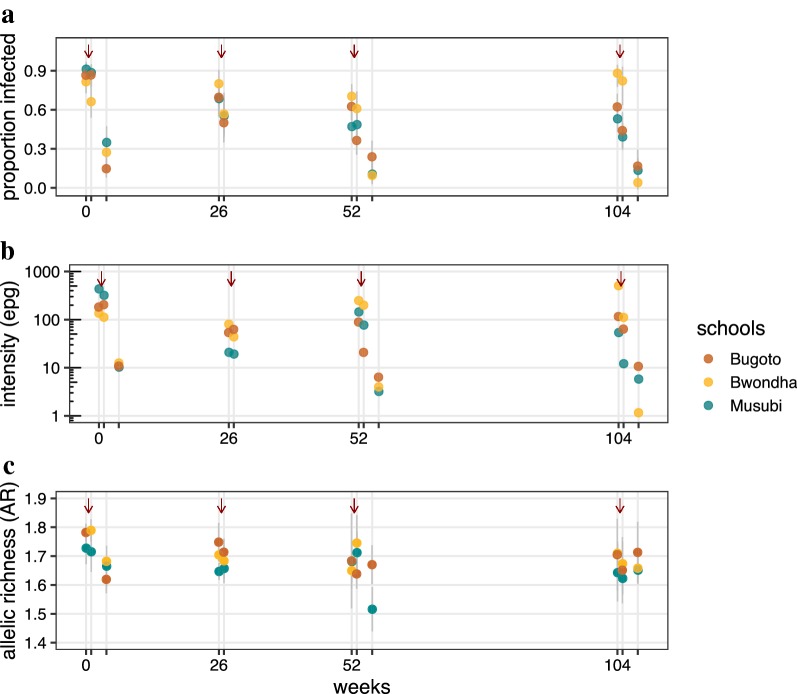
Epidemiology and genetic diversity of *S. mansoni* in Mayuge District from 2004–2006. Prevalence of *S. mansoni* infection (**a**) and mean infection intensity, as eggs per gram of stool (epg) (**b**), estimated with three days of replicate Kato-Katz in each of the three schools sampled. Red arrows indicate timing of mass praziquantel treatment. **c** The mean allelic richness for all infrapopulations sampled at each timepoint for each school. No miracidia were isolated from infrapopulations in Bwondha at week 56

The entire cohort was treated with praziquantel after each medium-term timepoint (no praziquantel treatment in the previous four weeks), indicated by red arrows in Figs. 1c and 2. At week 1, children with infection intensities greater than 100 epg were retreated with praziquantel. At all other timepoints, children were retreated with praziquantel if they had any *S. mansoni* eggs detected in any Kato-Katz slides. Children were treated with 40 mg/kg praziquantel, determined by weight. At all timepoints, observed treatment was recorded for each child.

A randomly selected subset of miracidia from 11 children at 26 and 27 weeks were used for an *in vitro* assay that measured phenotypic praziquantel susceptibility of miracidia. This *in vitro* assay exposes miracidia to praziquantel and uses changes in shape, behaviour and activity levels as a proxy for susceptibility and has been validated in prior laboratory and field studies [40, 45]. Here we have linked key summary measures for each infrapopulation to genetic data from these corresponding hosts. Two measures that showed the most variation between individuals (but not among) were used as a proxy for praziquantel susceptibility of miracidia: the proportion of miracidia that had abnormal movement and the proportion of miracidia that were immobile/dead after seven minutes *in vitro* exposure to 2 × 10^−6^ M praziquantel. Resistant genotypes have fewer changes in shape and behavioural responses to praziquantel exposure and thus a higher proportion of these miracidia are still behaving normally at seven minutes [40, 45]. Because individual miracidia were not simultaneously phenotyped and genotyped, average metrics for infrapopulations at each timepoint were linked.

### Laboratory analyses

DNA extraction and microsatellite analysis followed established protocols [44]. Briefly, individual miracidia were sized at seven microsatellite loci (Additional file 1: Table S3) in a single multiplex reaction that have low error rates in *S. mansoni* from Lake Albert, Uganda. Allele sizes were determined using ABI PRISM Genescan v2.7 and Genotyper v2.7 software (Applied Biosystems, Foster City, CA, USA).

While we aimed to amplify all microsatellites from 30 miracidia per infrapopulation per timepoint, one quarter of timepoints were represented by fewer than ten miracidia. This disparity in terms of sample size could affect the statistical power of the models and the accuracy of genetic diversity measures. However, a simulation study using similar microsatellite markers reported that more robust measures of genetic diversity are obtained when increasing the number of hosts rather than the number of miracidia per host [37]. Additional limitations of this study include genotypic errors inherent in these microsatellite markers [44] that may affect the conclusions. However, we were very stringent with allele calls and inclusion criteria to minimize these biases.

### Data analyses

All analyses were carried out in R v3.5.1 [46]. Specific packages are cited alongside functions used and summary code for these analyses can be found on github (see section “Availability of data and materials” below).

#### Epidemiological summary statistics

Individual schistosome infection intensities were calculated as an arithmetic mean of epg estimates from daily Kato-Katz slides examined at that timepoint. Paired rank sum Wilcoxon tests were used to test differences between pre- and post-treatment infection intensities. Population prevalence was calculated at each timepoint for each school and 95% confidence intervals (CI) intervals were calculated with Agresti-Coull approximations [47].

#### Genetic diversity measures

Departure from Hardy–Weinberg equilibrium (HWE) was quantified in *pegas* v0.11 [48], implementing the Monte Carlo procedure present in the function *hw.test* with 1000 permutations. At each sampling point, infrapopulation schistosome diversity observed heterozygosity (H_o_) and expected heterozygosity (H_e_) were calculated in *poppr* v2.8.1 [49]. Allelic richness (AR), which corrects the number of alleles per locus for uneven sample size, was calculated for each infrapopulation and timepoint using the *hierfstat* package v0.04-22 [50].

#### Determining spatial, temporal and host effects on infrapopulation genetic diversity

To identify potential factors that affected the observed infrapopulation genetic diversity of parasites at a given timepoint, we constructed linear models using the function *lm*. Child ID (unique value identifying individual) was included as a random effect in a linear mixed effect model using *lme4* [51] to account for repeated samples from the same infrapopulation over time, but was found to be insignificant. Explanatory variables included age, sex of child, cumulative number of observed praziquantel treatments, time since last observed treatment (in weeks), infection intensity at that sampling timepoint, and weeks from the beginning of MDA in that community. Sampling timepoints were also divided into three distinct categories: pre-treatment (weeks 0, 26, 52, 104); one week post-treatment (weeks 1, 27, 53, 58); and four weeks post-treatment (weeks 4, 56, 108). This was performed to increase statistical power, as post-treatment, especially at four weeks post-treatment, fewer miracidia were collected. The number of miracidia per infrapopulation per timepoint was included in the models as weights to reduce bias associated with estimates based on smaller sample sizes. Model comparison and selection was conducted using Akaike’s information criterion (AIC) [52].

#### Within-host dynamics

Some infrapopulations were sampled for miracidia at more than one timepoint. To further examine within-host dynamics, the genetic dataset was subset to include infrapopulations that were sampled at more than one timepoint, particularly pre-treatment and one week and four weeks post-treatment. Trees of infrapopulations over time were constructed using Nei’s distances in *poppr* v2.8.1 [49]. COLONY software was used to identify full-sibling pairs between miracidia within infrapopulations using the full likelihood method and long runs [53]. Because only seven microsatellite loci are used, the ability to detect half-siblings amongst this dataset was very limited and therefore the mating system was assumed to be monogamous. Miracidia with ≥ 0.75 probability of belonging to a family were included in the analysis (< 0.75 probability were assumed to be singletons). Our interest was to identify the occurrence of siblings between pre- and post-treatment sampling points, suggesting adult worms surviving treatment and reproducing viable offspring.

#### Quantifying population structure and gene flow

To determine the levels of gene flow, we used several methods to quantify structure of populations. Analysis of molecular variance (AMOVA), which detects population differentiation, was conducted using the function *amova* in *poppr* [49]. An AMOVA was carried out on the entire dataset to measure the genetic differentiation between schools, among children between schools and within children. AMOVAs were also carried out at each timepoint. *P*-values were calculated by 1000 random permutations. Population structure was also investigated using the discriminant analysis of principal components (DAPC) method [54] implemented in *adegenet* v2.1.1 [55], and by visualising Cavalli-Sforza & Edwards chord distances in *hierfstat* v0.04-22 with the neighbour-joining method implemented in *ape* v5.2 [56]. Phylogenetic trees were created using *in vitro* praziquantel data to elucidate whether infrapopulations with more drug-resistant phenotypes at that timepoint were genetically distinct from those infrapopulations which were more susceptible.

## Results

A total of 468 unique children were sampled for *S. mansoni* over 11 timepoints during the two-year study (Additional file 1: Table S2). Miracidia were isolated and analysed from 207 of these children from at least one timepoint. Deviations from Hardy–Weinberg equilibrium (HWE) were tested on the whole dataset of 4743 miracidia. The majority of infrapopulations at each timepoint were found to strongly deviate from HWE. We then excluded miracidia that were not genotyped at all seven microsatellite loci, leaving a total dataset of 3576 from 203 children (Fig. 1c). Despite a smaller total sample size, this subset showed little deviation from HWE and only four children were removed from the genetic analyses. The number of miracidia successfully genotyped at seven loci within an infrapopulation ranged from 1 to 94 per timepoint (mean 25.3).

### Baseline *S. mansoni* infections and genetic diversity

*Schistosoma mansoni* was found in 85.7% of individuals surveyed at the beginning of the study, indicating a high endemic transmission setting (Fig. 2a). The average infection intensity within an individual at the beginning of the study was 224.9 epg (moderate infection intensity; Fig. 2b). Genetic diversity of infrapopulations was also very high (Fig. 2c): average gene diversity among the loci (Hs) was 0.701 (range 0.280–0.888), while gene diversity among all populations (Ht) was 0.711 (range 0.282–0.901) (Additional file 1: Table S4). The number of alleles per locus ranged from 20 to 48. This supports the hypothesis that genetic diversity is high within these populations of *S. mansoni.*

### Effect of praziquantel treatment on *S. mansoni*

One week post-treatment, genetic diversity was not significantly different than pre-treatment. This was also reflected in some of the epidemiological data; at a majority of time points and schools, the prevalence and infection intensity at one week post-treatment were not significantly different to pre-treatment (Additional file 1: Tables S5, S6).

**Fig. 3:**
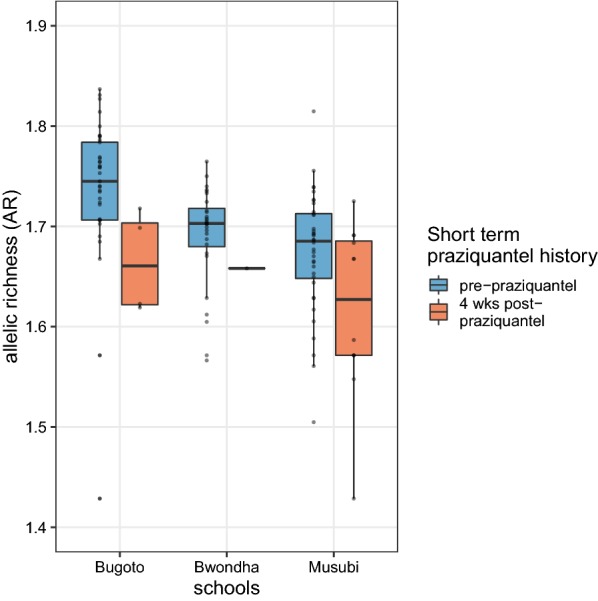
Short-term declines in genetic diversity between pre- and 4 weeks post-treatment. Boxplots of infrapopulation mean allelic richness are shown for each primary school averaged pre-praziquantel timepoints (weeks 0, 26, 52, 104) and compared to 4 weeks after praziquantel treatment (weeks 4, 56, 108). Note the smaller sample size post treatment due to the lower number of individuals shedding miracidia

However, prevalence and mean infection intensity significantly decreased at every four weeks post-treatment observation compared to pre-treatment (Fig. 2a, Additional file 1: Tables S5, S6). These data indicate a high level of success by praziquantel in reducing egg output four weeks after treatment and suggest there should be high levels of selection imposed on the parasites in treated infrapopulations. Concurrent to these epidemiological metrics, mean infrapopulation genetic diversity also significantly declined four weeks after each of the cohort treatments when accounting for age and sex of hosts (Fig. 3). This supports the hypothesis that praziquantel treatment reduces genetic diversity within treated individuals in the short-term following treatment.

Although these short-term effects were significant, prevalence, intensity and genetic diversity recovered over timescales greater than four weeks. Genetic diversity declined from baseline (week 0) to subsequent pre-treatment samples (six months, one year and two years), but this decline was not significant (*P *> 0.05). This supports the hypothesis that genetic diversity of *S. mansoni* is resilient to praziquantel, at least within this observation period and setting.

### Impact of treatment and host characteristics on *S. mansoni* infrapopulation genetic diversity

**Fig. 4:**
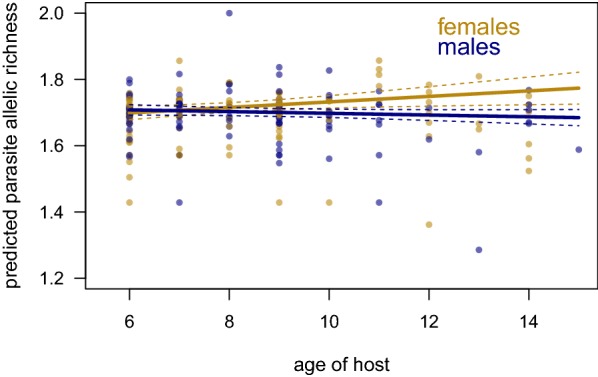
Genetic diversity of infrapopulations by age and sex. Regression lines are based on individuals pre-treatment and with no detectable eggs. Female infrapopulation genetic diversity increases with age (yellow), whereas male infrapopulation genetic diversity begins higher and decreases slightly with age (navy)

The best-fit model to explain infrapopulation genetic diversity, measured by AR at a given timepoint, contained infection intensity, short-term treatment, and an interaction between age and sex as significant predictor variables (Fig. 4). The mean AR of an infrapopulation for a female host pre-treatment, also the intercept in this model, was 1.65 (95% confidence interval (CI): 1.59 to 1.70). Each week post-treatment (up to four weeks), infrapopulation genetic diversity decreased (− 0.007, 95% CI: − 0.018 to − 0.001). Higher infection intensities slightly, but significantly, had higher genetic diversity; each additional 100 epg increased the mean allelic richness by 0.001 (95% CI: 0.0004 to 0.0020). Infrapopulations of males had a higher genetic diversity than those of females (0.07; 95% CI: 0.01 to 0.14). *Schistosoma mansoni g*enetic diversity in female hosts increased with age (0.008; 95% CI: 0.002 to 0.015); however, in male hosts the interaction between age and sex reduced genetic diversity (− 0.010; 95% CI: − 0.018 to − 0.003).

### Parasite structure and survival following treatment

Trees obtained from the Cavalli-Sforza & Edwards chord distances showed no clear clustering of infrapopulations between timepoints, suggesting no selection on these markers imposed by praziquantel treatment in the observed timeframe. Parasites excreted four weeks post-treatment were not more similar to one another compared with parasites collected pre-treatment within the same year and across all timepoints (Additional file 1: Figure S1). Additionally, infrapopulations with higher levels of *in vitro* drug-resistant phenotypes were not genetically distinct from infrapopulations with lower measures of drug-resistant phenotypes (Additional file 1: Table S7, Figure S2).

**Fig. 5:**
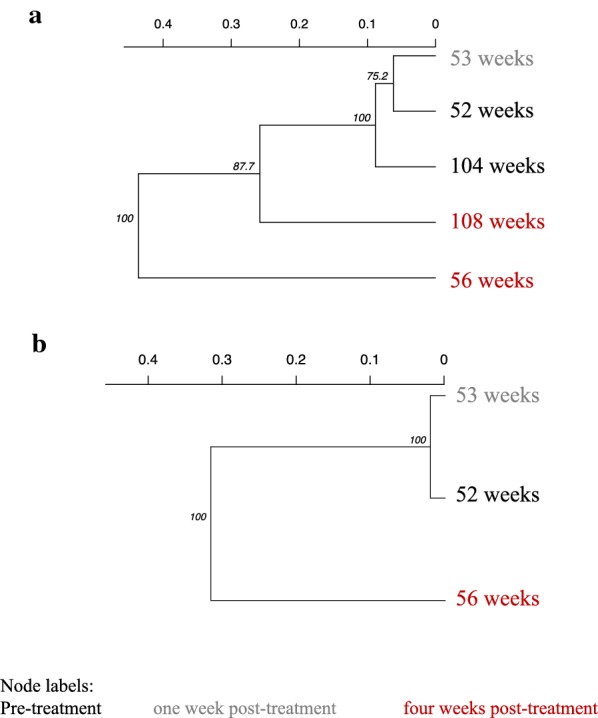
Phylogenies of infrapopulations from individual children sampled over time. The bootstrap support for each node is given and tips are labelled as the sampling time point. **a** A praziquantel naïve 6 year-old recruited in 2005 (52 weeks) in Musubi and followed-up at each time point afterwards. **b** A praziquantel naïve 6 year-old in 2005 (52 weeks) in Musubi that was followed at two post-treatment time point

**Fig. 6:**
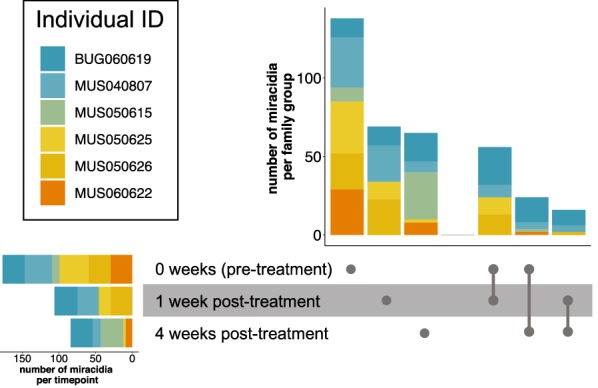
Histograms of family structure of full-siblings from six infrapopulations sampled pre-treatment, one week post-treatment and four weeks post-treatment. The distribution of miracidia from reconstructed maximum likelihood families are shown in the histogram. The majority of miracidia are from single families or families from the same timepoint; however, some full siblings are found between pre- and post-treatment, with the highest frequency between pre-treatment and one week post-treatment

Miracidia that were collected from six infrapopulations before and after treatment showed evidence for clustering of pre-treatment and one week post-treatment (Fig. 5, Additional file 1: Figure S3). Parasites four weeks post-treatment were more distant, even compared to parasites sampled pre-treatment a year apart. We used COLONY to detect full siblings within these infrapopulations sampled both pre- and post-treatment. Analysis of miracidia from these infrapopulations identified siblings between pre- and post-treatment sampling points (Fig. 6), suggesting adult worm pairs survived treatment and produced viable miracidia, particularly when siblings were found four weeks post-treatment. There was a higher proportion of siblings detected at one week post-treatment compared to four weeks post-treatment, which was supported by phylogenies by timepoint (Fig. 5), but this is confounded by lower numbers of miracidia recovered four weeks post-treatment. Full siblings were found up to one year apart and following praziquantel treatment (Additional file 1: Tables S8, S9), but the number of miracidia recovered over longer timescales is very limited.

### Gene flow between populations

The results of the AMOVA suggest a lack of structure between schools at different time points, showing that most of the variance in the dataset was explained by differences within hosts. Mean percentage of variation was 98.1% within hosts, 1.6% among hosts within schools and 0.3% between schools (Additional file 1: Table S10). F_ST_ was lower between schools, ranging from − 0.007 to 0.047, in the middle within schools, from 0.013 to 0.042 and higher within hosts, ranging from 0.015 to 0.088 (Additional file 1: Figure S4). The tree obtained from the Cavalli-Sforza & Edwards chord distances showed no clear clustering between villages (Additional file 1: Figure S5). Clustering algorithms implemented in DAPC also failed to identify an informative number of clusters in relation to school or timepoint (Additional file 1: Figure S6). These results support the hypothesis that gene flow is high between these populations.

## Discussion

Using longitudinal epidemiological and genetic data from the start of MDA in Uganda, we show there are short-term effects of praziquantel treatment on *S. mansoni* but populations recover within six months. Although we identify parasites surviving treatment even at the beginning of MDA, there is no evidence that these parasites are selected for over this two-year period. High rates of gene flow between populations and refugia in snails and untreated individuals likely facilitate rapid recovery of parasite genetic diversity and prevent fixation of resistant/tolerant parasites.

Cure rates during this study were within praziquantel expectations at a population level for *S. mansoni* [57], suggesting that resistant/tolerant parasites are not overabundant in these study populations. However, we observed adult worms surviving treatment, as evidenced by full siblings found pre- and four weeks post-treatment in four of six infrapopulations with sufficient sampling frequency. We believe these are resistant or tolerant adult worms and are likely a subset of the natural variation (rather than a result of selection). Infrapopulation genetic diversity was significantly lower at four weeks post-treatment, supporting this idea. Juveniles at point of treatment could be contributing to some eggs observed at four weeks post-treatment, but the presence of siblings pre- and four weeks post-treatment suggest that at least some eggs are from adult worms that survive praziquantel. We also observed variation in phenotypic praziquantel susceptibility, but we did not directly sequence these parasites. The phenotypic and genetic data from this setting suggest that natural variation in this schistosome population has some praziquantel resistance or tolerance (we could not differentiate these with our data). This is consistent with evidence of natural variation in resistance within schistosomes predating drug use to a former anti-schistosomal drug, oxaminiquine, where resistance alleles are known [58].

Despite evidence of resistant/tolerant parasites in this population, there was no evidence for selection for these parasites in the timeframe observed. Parasites found four weeks after treatment did not cluster, nor did phenotypically resistant parasite populations. High rates of transmission and high rates of gene flow likely prevent population bottlenecking and could reduce the likelihood of resistance developing at a local level at the coverage levels and short- to medium- (under two years) time scales studied here [59]. Our genetic markers are likely not reflective of resistance; these microsatellite markers do not map to population (our study) or individual phenotypes [60]. It is not expected that microsatellites would be accurate markers for resistance, unless they were located physically close to praziquantel-resistant genes (which are not yet characterised in any *Schistosoma* species). Although these microsatellites do not seem to serve as resistance markers, they are useful for parentage analysis and identifying worms that survive treatment. Despite no evidence for selection in this study, concerted drug treatment across the area for several years may have selected for these resistant worms over longer timescales and resulted in the low cure rates currently observed in the region more recently [24].

Interestingly, there was very little difference in genetic diversity between pre- and one week post-treatment. We expect this is because eggs were still being excreted from adult worms that had produced the eggs before treatment, but which may have then died with treatment. Because genetic diversity and infection intensity were significantly lower at four weeks post-treatment, we expect most eggs of susceptible worms to be expelled by four weeks post-treatment. It is thought that eggs only survive up to three weeks after expulsion from the female [61]. This is supported by sibship analysis that finds a higher frequency of siblings between pre- and one week post-treatment compared to four weeks post-treatment. It is important to note that the majority of the host population are still shedding viable eggs one week post-treatment, meaning hosts contribute to transmission even a week after successful treatment. Infection intensities one week post-treatment in some schools and some timepoints were not significantly different from pre-treatment infection intensities, further emphasizing the potentially significant contribution to transmission in these communities.

There was no strong evidence for effects of praziquantel on genetic diversity over the medium-term. This is supported by other studies across sub-Saharan Africa [2, 26, 30, 60, 62]. Most studies focus on periods well after national control programmes begin. Only one other study, in addition to this one, examines parasite diversity and structure at the start of MDA. Norton et al. [21] found an initial decline six months after treatment at the start of MDA in Tanzania; however, a follow-up five years later demonstrated that parasite genetic diversity had recovered and even increased in these same schools [60]. One explanation for an initial decline in genetic diversity observed in Tanzania is a higher degree of population structure among the parasites compared to our sites. We did not observe genetic diversity declines after six months (only four weeks post-treatment), but recovery of genetic diversity of parasites at these Ugandan schools may be facilitated by higher rates of gene flow. Post-treatment parasite populations are small in comparison to the refugia in untreated humans within the community and other contributing communities as well as parasites in snails [63, 64]. Combined, these studies emphasize the resilience of schistosome parasite populations to repeated praziquantel treatments.

Many studies, including ours, find that the majority of the genetic diversity in *S. mansoni* occurs at the human host level, rather than village or district level [21, 28, 29, 31, 33]. This can be explained by a limited number of shared water contact sites and/or cercariae dispersing far enough to cover these sites. Genetic diversity was not significantly different between villages, suggesting similar exposure environments (all are along Lake Victoria) and further supporting a panmictic parasite population across the area surveyed. We also observed high levels of genetic diversity, similar to other studies that examined *S. mansoni* populations in Uganda [31, 65], and higher than that reported from other localities in East Africa. For example, a study focusing on four villages in Ethiopia [66] reported a total of six and 15 alleles for the SMD28 and SMDA28 loci, while in this study we recovered 26 and 54 alleles for the two loci, respectively. Lake Victoria is likely the origin of *S. mansoni* and larger scale surveys have reported the area to have the highest levels of genetic diversity across several markers [24, 31, 65, 67]. This high genetic diversity can increase chances of drug resistance developing and also help these populations recover from bottlenecking selection [12, 13]. However, high genetic diversity can also reduce the likelihood of an allele fixing in a population and can prevent resistance from spreading.

We found that infrapopulation genetic diversity is also significantly related to host age and sex. We interpret infrapopulation genetic diversity as the combined outcome of the genetic diversity of parasites circulating in the environment, variation in behaviour (particularly those related to water contact, i.e. location, duration and time of day), and establishment probability (dependent on host susceptibility and immune history and parasite infectiousness). We found that males had higher parasite genetic diversity than females. We expect this higher genetic diversity, particularly at younger ages, to reflect a difference in behaviour as young males have reported to play in the water more often than females of a similar age [68]. This effect of sex was dependent on age; males had similar genetic diversity across all ages surveyed here whereas each additional year significantly increased the genetic diversity of parasites observed in females. Older females (i.e. 10 years-old and above) help more in household chores like clothes washing and fetching water [68], which would increase their exposure to schistosomes and likely increase the genetic diversity observed in an infrapopulation. A study of *S. haematobium* in Mali also found significant impacts of host demographics; males had more unique genotypes and these private alleles increased with age [69]. Neither study, however, observed any decline in genetic diversity with age, as may be expected if immunity was developing. It may be that the genetic diversity is very high in these settings and new genotypes are constantly being encountered which hosts have not yet acquired immunity against. An alternative, non-exclusive, explanation is that the ages sampled here (6–14 years) are insufficient to detect this effect of an immune response on genetic diversity.

## Conclusions

This study highlights the resilience of schistosome populations to repeated drug treatments in a high endemicity setting in Uganda. We found evidence for adult worms surviving treatment at the beginning of the national control programme, suggesting natural variation in resistance/tolerance. These may have been selected on with subsequent round of MDA and led to low cure rates observed a decade later. In settings with similar epidemiology and genetic diversity as observed here, MDA alone is unlikely to be sufficient for elimination and could even lead to long-term issues if drug resistance is selected.

## Supplementary information

**Additional file 1: Text S1.** Additional methods. **Table S1.** Travel distances between schools. **Table S2.** Summary of infrapopulations (individuals) sampled at each timepoint (listed as weeks since start of study). **Table S3.** Microsatellite loci utilized in this study. **Text S2.** Additional results. **Table S4.** Comparison of genetic diversity at the seven microsatellite loci used in the study. **Table S5.** Proportion infected at pre- and post-treatment. **Table S6.** Infection intensity between pre- and post-treatment timepoints. **Table S7.** Summary of infrapopulation phenotyped. **Table S8.** Frequency of miracidia isolated per individual from relative timepoints. **Table S9.** Frequency of full-sibling miracidia belonging to a family structure (including singletons) per individual. **Table S10.** Analysis of molecular variance (AMOVA) for *Schistosoma mansoni*. **Figure S1.** Parasite structure through time. **Figure S2.** Trees of phenotype data. **Figure S3.** Phylogenies of infrapopulations from individual children sampled over time (continued from Fig. 5). **Figure S4.** Pairwise F_ST_ between each school and timepoint. **Figure S5.** Population differentiation by village. **Figure S6.** Clustering by discriminant analysis of principal components (DAPC).

## Data Availability

Archived raw data are deposited at researchdata.gla.ac.uk (10.5525/gla.researchdata.931). Code is available at https://github.com/cfaustus/ug_pop_gen_2004_2006.

## Abbreviations

AIC: akaike’s information criterion
AMOVA: analysis of molecular variance
CI: confidence interval
DAPC: discriminant analysis of principal components
epg: eggs per gram
HWE: Hardy–Weinberg equilibrium
MDA: mass drug administration
WASH: water, sanitation and hygiene
