# Supplementary material for: Two-year longitudinal survey reveals high genetic diversity of Schistosoma mansoni with adult worms surviving praziquantel treatment at the start of mass drug administration in Uganda
Source: Parasit Vectors. 2019 Dec 27;12:607. doi: 10.1186/s13071-019-3860-6 (PMC6935072; doi:10.1186/s13071-019-3860-6)
Supplement: Supplementary file 1 — Additional file 1: Text S1. Additional methods. Table S1. Travel distances between schools. Table S2. Summary of infrapopulations (individuals) sampled at each timepoint (listed as weeks since start of study). Table S3. Microsatellite loci utilized in this study. Text S2. Additional results. Table S4. Comparison of genetic diversity at the seven microsatellite loci used in the study. Table S5. Proportion infected at pre- and post-treatment. Table S6. Infection intensity between pre- and post-treatment timepoints. Table S7. Summary of infrapopulation phenotyped. Table S8. Frequency of miracidia isolated per individual from relative timepoints. Table S9. Frequency of full-sibling miracidia belonging to a family structure (including singletons) per individual. Table S10. Analysis of molecular variance (AMOVA) for Schistosoma mansoni. Figure S1. Parasite structure through time. Figure S2. Trees of phenotype data. Figure S3. Phylogenies of infrapopulations from individual children sampled over time (continued from Fig. 5). Figure S4. Pairwise FST between each school and timepoint. Figure S5. Population differentiation by village. Figure S6. Clustering by discriminant analysis of principal components (DAPC). [file 13071_2019_3860_MOESM1_ESM.docx]

**ADDITIONAL FILE 1**

**Text S1.** ADDITIONAL METHODS

*Recruitment of samples.* Children were initially recruited at Bwondha Primary School and Bugoto Lake View Primary School by the Schistosomiasis Control Initiative in 2003. Miracidia from these individuals were not stored for genetic analysis, but their treatment history was recorded. Musubi Church of God Primary School was first treated in this study, and to our knowledge the majority of children were praziquantel-naïve prior to 2004 (20 children ~11 year old were treated in 2003 [1]– but unlikely to be recruited in this study).

**Table S1. Travel Distances between schools.** Approximate distances by road and shorelines are given between each primary school. To translate into travel time, road travel between Musubi Church of God and Bwondha Primary School takes approximately 80 minutes in the dry season in a 4-wheel drive vehicle.

|  | Bugoto Lake View | | Bwondha | | Musubi Church of God | |
| --- | --- | --- | --- | --- | --- | --- |
|  | **road** | **shoreline** | **road** | **shoreline** | **road** | **shoreline** |
| Bugoto Lake View | - | - | 36.5 km | 34.7 km | 10.1 km | 7.7 km |
| Bwondha | 36.5 km | 34.7 km | - | - | 41.6 km | 42.4 km |
| Musubi Church of God | 10.1 km | 7.7 km | 41.6 km | 42.4 km | - | - |

**Table S2. Summary of infrapopulations (individuals) sampled at each timepoint (listed as weeks since start of study).** The numbers indicate total unique individuals with at least one Kato-Katz slide. Numbers in red are the total individuals that are praziquantel naïve (have never had treatment in their lifetime).

| School | 2004 | | | | | 2005 | | | 2006 | | |
| --- | --- | --- | --- | --- | --- | --- | --- | --- | --- | --- | --- |
|  | **0** | **1** | **4** | **26** | **27** | **52** | **53** | **56** | **104** | **105** | **108** |
| Bugoto Lake View | 104 (21) | 75 | 75 | 46 | 40 | 96 (33) | 66 | 63 | 82 (25) | 50 | 54 |
| Bwondha | 108 (28) | 68 | 66 | 35 | 30 | 81 (30) | 46 | 42 | 67 (28) | 28 | 25 |
| Musubi Church of God | 68 (68) | 61 | 66 | 54 | 47 | 85 (31) | 70 | 76 | 100 (30) | 87 | 82 |

**Table S3. Microsatellite loci utilized in this study.**

| Locus | Reference | Genbank Acc no. | Repeat motif | Size range (bp) |
| --- | --- | --- | --- | --- |
| SMDA28 | Curtis et al. (2001) | AF325695 | GATA | 92–128 |
| SMD25 | Durand et al. (2000) | AF202965 | CA | 272–312 |
| SMD28 | Durand et al. (2000) | AF202966 | CAA | 230–245 |
| SMD89 | Durand et al. (2000) | AF202968 | TC | 138–169 |
| SMU31768 | Durand et al. (2000) | U31768 | GAT | 179–247 |
| CA11-1 | Blair et al. (2001) | AI068336 | GA, GT | 191–231 |
| SMS9-1 | Blair et al. (2001) | AF330106 | GT | 178–208 |

Text S2. ADDITIONAL RESULTS

The results are for the truncated dataset that includes children from three schools: 69 hosts and 1,399 miracidia from Bugoto, 58 and 993 for Bwondha, and 76 and 1,184 for Musubi. No samples were recovered from Bugoto at week one, and no miracidia were successfully genotyped for Bwondha at week 56. A total of 19 children had only one miracidium genotyped, and 65 children had less than ten.

**Table S4. Comparison of genetic diversity at the seven microsatellite loci used in the study.** Na = allele number, Hexp is Nei's 1978 gene diversity, and evenness is a metric of the distribution of genotypes. An evenness score of 0 is an uneven distribution (dominated by a single genotype) wheres as closer to 1 represents equal abundances of all genotypes.

| **Locus** | **Na** | **Hexp** | **evenness** |
| --- | --- | --- | --- |
| SMDA28 | 46 | 0.90 | 0.79 |
| SMD25 | 29 | 0.80 | 0.70 |
| SMD28 | 24 | 0.32 | 0.37 |
| SMD89 | 20 | 0.64 | 0.68 |
| SMU31768 | 48 | 0.85 | 0.75 |
| CA11-1 | 33 | 0.82 | 0.73 |
| SMS9-1 | 37 | 0.68 | 0.51 |

**Table S5. Proportion infected at pre- and post- treatment sampling points**

| **school** | **‘pre’- treatment week** | **post- treatment week** | **pre- treatment proportion infected** | **post- treatment proportion infected** | **test statistic** | **P- value** |
| --- | --- | --- | --- | --- | --- | --- |
| Bugoto | 0 | 1 | 0.87 | 0.87 | 0.0000 | 1.0000 |
| Bugoto | 0 | 4 | 0.87 | 0.15 | 88.6482 | 0.0000 |
| Bugoto | 26 | 27 | 0.70 | 0.50 | 2.6565 | 0.1031 |
| Bugoto | 52 | 53 | 0.63 | 0.36 | 9.6803 | 0.0019 |
| Bugoto | 52 | 56 | 0.63 | 0.24 | 21.3233 | 0.0000 |
| Bugoto | 104 | 105 | 0.62 | 0.44 | 3.4565 | 0.0630 |
| Bugoto | 104 | 108 | 0.62 | 0.17 | 25.5590 | 0.0000 |
| Musubi | 0 | 1 | 0.91 | 0.89 | 0.0427 | 0.8363 |
| Musubi | 0 | 4 | 0.91 | 0.35 | 43.4170 | 0.0000 |
| Musubi | 26 | 27 | 0.69 | 0.55 | 1.3455 | 0.2461 |
| Musubi | 52 | 53 | 0.47 | 0.49 | 0.0007 | 0.9792 |
| Musubi | 52 | 56 | 0.47 | 0.11 | 23.8755 | 0.0000 |
| Musubi | 104 | 105 | 0.53 | 0.39 | 3.0853 | 0.0790 |
| Musubi | 104 | 108 | 0.53 | 0.13 | 29.2546 | 0.0000 |
| Bwondha | 0 | 1 | 0.81 | 0.66 | 4.4976 | 0.0339 |
| Bwondha | 0 | 4 | 0.81 | 0.27 | 48.3126 | 0.0000 |
| Bwondha | 26 | 27 | 0.80 | 0.57 | 3.1060 | 0.0780 |
| Bwondha | 52 | 53 | 0.70 | 0.61 | 0.8057 | 0.3694 |
| Bwondha | 52 | 56 | 0.70 | 0.10 | 38.5650 | 0.0000 |
| Bwondha | 104 | 105 | 0.88 | 0.82 | 0.1915 | 0.6616 |
| Bwondha | 104 | 108 | 0.88 | 0.04 | 53.0672 | 0.0000 |

**Table S6. Infection Intensity between pre- and post-treatment timepoints.** Significant declines were tested using a one-sided Wilcoxan Rank Sign Test.

| **school** | **‘pre’- treatment week** | **post- treatment week** | **pre- treatment mean infection intensity** | **post- treatment mean infection intensity** | **test statistic** | **P- value** |
| --- | --- | --- | --- | --- | --- | --- |
| Bugoto | 0 | 1 | 204.92 | 203.06 | 1427 | 0.1408 |
| Bugoto | 0 | 4 | 186.04 | 9.84 | 2434 | 0.0000 |
| Bugoto | 26 | 27 | 52.20 | 61.62 | 188 | 0.5144 |
| Bugoto | 52 | 53 | 78.09 | 19.82 | 989 | 0.0000 |
| Bugoto | 52 | 56 | 61.10 | 5.26 | 862.5 | 0.0000 |
| Bugoto | 104 | 105 | 86.58 | 62.60 | 475 | 0.0012 |
| Bugoto | 104 | 108 | 81.58 | 9.67 | 737.5 | 0.0000 |
| Bwondha | 0 | 1 | 137.19 | 111.22 | 1300.5 | 0.0009 |
| Bwondha | 0 | 4 | 128.22 | 11.53 | 1495 | 0.0000 |
| Bwondha | 26 | 27 | 71.27 | 42.87 | 256 | 0.0548 |
| Bwondha | 52 | 53 | 210.27 | 198.56 | 350 | 0.1870 |
| Bwondha | 52 | 56 | 212.09 | 3.00 | 389 | 0.0000 |
| Bwondha | 104 | 105 | 156.02 | 113.88 | 238.5 | 0.0561 |
| Bwondha | 104 | 108 | 156.03 | 0.16 | 276 | 0.0000 |
| Musubi | 0 | 1 | 448.08 | 318.95 | 1102 | 0.0010 |
| Musubi | 0 | 4 | 442.23 | 9.28 | 1888 | 0.0000 |
| Musubi | 26 | 27 | 19.66 | 18.24 | 326 | 0.3155 |
| Musubi | 52 | 53 | 162.40 | 75.97 | 635.5 | 0.0040 |
| Musubi | 52 | 56 | 148.64 | 2.24 | 672.5 | 0.0000 |
| Musubi | 104 | 105 | 39.51 | 11.13 | 831.5 | 0.0063 |
| Musubi | 104 | 108 | 46.36 | 4.80 | 949 | 0.0000 |

*Phenotyping data.* Two measures were used as a proxy for praziquantel susceptibility of miracidia: the proportion of miracidia that had abnormal movement and the proportion of miracidia that were immobile/dead after seven minutes *in vitro* exposure to 2 × 10^−6^ M praziquantel. 799 miracidia from 11 children at 26 weeks (6 months) and 27 weeks (6 months, 1 week) were used for the genetic analysis.

**Table S7. Summary of infrapopulations phenotyped.** For the phenotyping assay, the school, unique child ID, timepoint (either 26 or 27 weeks – pre and one week post treatment), phenotype data and number of miracidia analysed for microsatellites are given.

| school | full unique child ID | Timepoint  (Pre = 26 weeks; Post = 27 weeks) | miracidia phenotyped | percent dead at 7 minutes | percent abnormal at 7 minutes | miracidia genotyped for microsatellites |
| --- | --- | --- | --- | --- | --- | --- |
| Bugoto | BUG030609 | Pre | 18 | 72 | 94 | 93 |
| Bugoto | BUG030632 | Post | 24 | 17 | 96 | 32 |
| Bugoto | BUG030704 | Pre | 21 | 71 | 95 | 94 |
| Bugoto | BUG030704 | Post | 24 | 21 | 100 | 89 |
| Bugoto | BUG030705 | Post | 22 | 0 | 100 | 74 |
| Bugoto | BUG031104 | Pre | 22 | 86 | 95 | 90 |
| Bugoto | BUG031104 | Post | 21 | 10 | 100 | 84 |
| Bwondha | BWD030840 | Pre | 20 | 25 | 100 | 30 |
| Bwondha | BWD031106 | Pre | 19 | 53 | 100 | 30 |
| Bwondha | BWD031106 | Post | 24 | 21 | 100 | 30 |
| Musubi | MUS040702 | Post | 24 | 67 | 100 | 32 |
| Musubi | MUS040708 | Pre | 22 | 36 | 86 | 31 |
| Musubi | MUS040811 | Pre | 14 | 43 | 100 | 30 |
| Musubi | MUS041208 | Pre | 7 | 29 | 100 | 30 |
| Musubi | MUS041208 | Post | 8 | 63 | 100 | 30 |

*Sib-ship analysis.* Six children were used for sibship analysis because miracidia were available for multiple sampling points pre- and post- treatment. Some of miracidia isolated from these individuals were from longer time scales (i.e. one year apart – MUS). The figure in the main text (Fig 6) details only sibling relationships between short timescales (up to one month apart). However, in Table S9 the inventory of what relative timepoint miracidia were isolated from is given and in Table S10 the miracidia are shown in their full-sibling family groupings.

**Table S8. Frequency of miracidia isolated per individual from relative timepoints.** Abbreviations are for relative sampling point: 0W (pre-treatment; i.e. no PZQ treatment in previous 4 weeks), 1W (one week post-treatment) and 4W (four weeks post-treatment).

| **unique ID** | **0W** | **1W** | **4W** | **1Y1W (prior)** | **1 Y (after)** | **1W4W**  **(after)** |
| --- | --- | --- | --- | --- | --- | --- |
| BUG060619 | 30 | 31 | 30 |  |  |  |
| MUS040807 | 38 | 29 | 11 |  |  |  |
| MUS050615 | 10 | 0 | 31 | 30 |  |  |
| MUS050625 | 30 | 30 | 1 |  | 6 | 1 |
| MUS050626 | 39 | 16 | 2 |  |  |  |
| MUS060622 | 30 | 0 | 9 |  |  |  |

**Table S9. Frequency of full-sibling miracidia belonging to a family structure (including singletons) per individual.** Columns in red are not shown in main text and represent full- siblings or singletons isolated more than four weeks apart.

| **unique ID** | 0W | 1W | 4W | 0W - 1W | 0W - 4W | 1W - 4W | 1Y1W (prior) | 1Y | 1Y4W | 1Y1W (prior) - 0W | 1Y1W (prior) - 4W | 0W - 1Y | 1W - 1Y |
| --- | --- | --- | --- | --- | --- | --- | --- | --- | --- | --- | --- | --- | --- |
| BUG060619 | 12 | 12 | 18 | 24 | 16 | 10 |  |  |  |  |  |  |  |
| MUS040807 | 32 | 23 | 7 | 8 | 4 | 4 |  |  |  |  |  |  |  |
| MUS050615 | 8 | 22 | 24 | 3 | 2 | 12 | 23 |  |  | 3 | 10 |  |  |
| MUS050625 | 32 | 10 | 2 | 11 | 0 | 0 |  | 3 | 1 |  |  | 2 | 3 |
| MUS050626 | 23 | 23 | 0 | 13 | 0 | 2 |  |  |  |  |  |  |  |
| MUS060622 | 29 | 0 | 8 | 0 | 2 | 0 |  |  |  |  |  |  |  |

**Table S10. Analysis of molecular variance (AMOVA) for Schistosoma mansoni.** Values are per sampling point between schools and hosts, and within hosts. P values were assessed using 1000 permutations.** indicates a p-value less than 0.5.

| **Week** | **Source of variation** | **degrees of freedom** | **Sum of squares** | **Variance components** | **Percentage of variation** | **P-value** |
| --- | --- | --- | --- | --- | --- | --- |
| 0 | Among schools | 2 | 9.89 | 0.0037 | 0.10 | 0.01** |
|  | Among hosts within schools | 9 | 43.09 | 0.0702 | 1.92 | 0.01** |
|  | Within hosts | 195 | 697.60 | 3.5774 | 97.98 | 0.38 |
| 1 | Among schools | 1 | 5.53 | 0.0294 | 0.79 | 0.02** |
|  | Among hosts within schools | 7 | 29.89 | 0.0541 | 1.46 | 0.04** |
|  | Within hosts | 107 | 387.97 | 3.6259 | 97.75 | 0.18 |
| 4 | Among schools | 2 | 7.76 | -0.0405 | -1.48 | 0.02** |
|  | Among hosts within schools | 4 | 12.77 | 0.0973 | 3.56 | 0.24 |
|  | Within hosts | 119 | 318.88 | 2.6796 | 97.92 | 0.55 |
| 26 | Among schools | 2 | 37.86 | 0.0545 | 1.73 | 0.01** |
|  | Among hosts within schools | 15 | 129.64 | 0.1762 | 5.58 | 0.01** |
|  | Within hosts | 582 | 1701.88 | 2.9242 | 92.69 | 0.11 |
| 27 | Among schools | 2 | 12.89 | 0.0146 | 0.50 | 0.01** |
|  | Among hosts within schools | 18 | 71.11 | 0.0441 | 1.52 | 0.01** |
|  | Within hosts | 521 | 1484.53 | 2.8494 | 97.98 | 0.01** |
| 52 | Among schools | 2 | 9.45 | 0.0013 | 0.04 | 0.01** |
|  | Among hosts within schools | 22 | 91.70 | 0.0608 | 1.93 | 0.01** |
|  | Within hosts | 436 | 1347.00 | 3.0894 | 98.03 | 0.33 |
| 53 | Among schools | 2 | 9.99 | 0.0169 | 0.56 | 0.03** |
|  | Among hosts within schools | 24 | 77.54 | 0.0228 | 0.75 | 0.09** |
|  | Within hosts | 294 | 875.88 | 2.9792 | 98.69 | 0.05** |
| 56 | Among schools | 1 | 4.60 | 0.2152 | 6.82 | 0.12 |
|  | Among hosts within schools | 3 | 9.73 | 0.0842 | 2.67 | 0.12 |
|  | Within hosts | 23 | 65.73 | 2.8577 | 90.52 | 0.09** |
| 104 | Among schools | 2 | 13.61 | 0.0106 | 0.34 | 0.01** |
|  | Among hosts within schools | 38 | 155.68 | 0.0702 | 2.28 | 0.01** |
|  | Within hosts | 616 | 1848.91 | 3.0015 | 97.38 | 0.02** |
| 105 | Among schools | 2 | 8.67 | 0.0045 | 0.15 | 0.01** |
|  | Among hosts within schools | 25 | 87.35 | 0.0450 | 1.52 | 0.01** |
|  | Within hosts | 356 | 1036.00 | 2.9101 | 98.33 | 0.1 |
| 108 | Among schools | 2 | 5.83 | -0.0316 | -1.08 | 0.06 |
|  | Among hosts within schools | 7 | 26.15 | 0.0735 | 2.51 | 0.02** |
|  | Within hosts | 124 | 358.06 | 2.8876 | 98.57 | 0.82 |

**Figure S1. Parasite structure through time**. Parasites isolated at certain timepoints and villages were not more similar to one another. Tree constructed using minimum spanning distances and implementing in poppr. A cutoff of 0.3 was used to improve visualization.

**Figure S2. Nei distance phylogenies of infrapopulation miracidia phenotypes. Nei distances and 10000 bootstraps. Only nodes supported by >75% of bootstrap runs are shown.**

a) Percentage of individual miracidia dead at 7 minutes (trials of 20-22 miracidia per infrapopulation table S3

b) proportion of miracidia abnormal shape at 7 minutes (majority were 100%)

**Figure S3.** **Phylogenies of infrapopulations from individual children sampled over time** (continued from Figure 5).

**Figure S4. Pairwise F_ST_ between each school and timepoint.**

**Figure S5. Population differentiation by village.** No clustering between villages from Bruvo or hamming distances.

**Figure S6.** **Clustering by Discriminant analysis of principal components (DAPC).** DAPC failed to identify an informative number of clusters in relation to A) school or B) timepoint.

A. Clustering by school: Musubi (MUS), Bugoto (BUG) and Bwondha (BWD) shows extensive overlap across timepoints (same when partitioned by time as well)

B. DAPC by timepoint (in weeks) Dark red earlier to yellow (2+ years) shows no structure temporally.

**References**:

1. Stothard JR, Kabatereine NB, Tukahebwa EM, Kazibwe F, Mathieson W, Webster JP, et al. Field evaluation of the Meade Readview handheld microscope for diagnosis of intestinal schistosomiasis in Ugandan school children. Am J Trop Med Hyg. 2005;73:949-55.
